# High-fat diet-associated cognitive decline: Is zinc finger protein 1 (ZPR1) the molecular connection?

**DOI:** 10.1016/j.crphys.2021.09.004

**Published:** 2021-10-02

**Authors:** Mythri Chittilla, Nuraly S. Akimbekov, Mohammed S. Razzaque

**Affiliations:** a Department of Pathology, Lake Erie College of Osteopathic Medicine, Erie, PA, USA; b Department of Biotechnology, Al-Farabi Kazakh National University, Almaty, Kazakhstan

**Keywords:** ZPR1, High-fat diet, Neuronal death, Cognitive function

## Abstract

Zinc finger protein 1 (ZPR1) is required for cellular replication and viability. Recently, ZPR1 variant rs964184 has been repeatedly linked to high plasma triglyceride levels, metabolic syndrome, type 2 diabetes mellitus (T2DM), and nonalcoholic fatty liver disease (NAFLD), suggesting its involvement in lipid metabolism. This article attempts to explain how ZPR1 contributes to the mechanism of high-fat diet-associated cognitive decline through three premises: i) high-fat diet results in cognitive decline, ii) ZPR1 deficiency also results in cognitive decline, and iii) high-fat diet results in ZPR1 deficiency. Therefore, ZPR1 has the potential to be the connection between high-fat diet and cognitive decline. The two modalities of cognitive decline caused by low concentrations of ZPR1 are reduced brain-derived growth factor (BDNF) synthesis and neuron death, both occurring in the hippocampus. Downregulation of ZPR1 may lead to decreased synthesis of BDNF due to reduced concentrations of peroxisome proliferator-activated receptor-gamma (PPAR-γ), tropomyosin receptor kinase B (Trk B), and cAMP response element-binding protein (CREB), resulting in reduced ability to form and retain long-term memory as well as reduced neuroplasticity. Likewise, low concentrations of ZPR1 facilitate neuron death by producing lower amount of spinal motor neuron (SMN) protein, causing genomic instability, activating mixed-lineage protein kinase 3 (MLK3), mitogen-activated protein kinase 7 (MKK7), and c-Jun N-terminal kinase 3 (JNK3) signal cascade, and ultimately resulting in the activation of Caspase 3.

## Highlights

• ZPR1 plays an important role in cell division and viability.
• ZPR1 variant rs964184 has been linked to dysregulation of lipid metabolism.
• High-fat diet can reduce ZPR1 to induce hippocampal neuronal death.
• High-fat diet correlates with cognitive decline.
• ZPR1 may mediate high-fat diet-induced cognitive decline.

## 1 Introduction

High-fat diets, containing high in saturated and trans fats, have been repeatedly linked to cognitive decline through the onset of Alzheimer's disease. Several models demonstrated high-fat diet-mediated cognitive decline, but none explained how zinc finger protein 1 (ZPR1) plays a role. Although ZPR1 is not the sole cause of Alzheimer's disease or vascular dementia, it may have a role in neuronal cell death and cognitive decline following a high-fat diet. This idea needs further validation by functional and clinical studies. This brief article explains the potential role(s) of ZPR1 in maintaining cognitive functions, in addition to its known functions in Spinal Muscular Atrophy (SMA).

ZPR1 is a highly conserved, ubiquitously expressed protein and is part of the zinc finger family (Galcheva-Gargova et al., 1996). Upon its discovery, it was quickly proven that its presence in the nucleus is vital for cellular replication and viability (Galcheva-Gargova et al., 1996). ZPR1 has many functions, including but not limited to: assembling into multiprotein complexes with survival motor neuron (SMN) protein as a part of RNA splicing (Mishra et al., 2007), binding to the promotor of *SMN 2*, binding to RNA polymerase (Kannan et al., 2018, Kannan et al., 2020), negatively regulating epidermal growth factor receptor (EGFR) (Galcheva-Gargova et al., 1996), positively regulating PPAR-γ 1/2 transcription (Corton et al., 2000; Mangelsdorf et al., 1995), and forming vital complexes with eukaryotic translation elongation factor 1A (eEF1A) in the GDP state (Mishra et al., 2007). ZPR1 is extensively studied because it heavily contributes to the underlying mechanism of SMA. Recently, it was discovered that the downregulation of ZPR1 in SMA contributes to neuron death (Ahmad et al., 2016). SMA is caused by mutations in *SMN1* and the phenotype is modified by the copy number of *SMN2*, as more SMN protein reduces the severity of SMA (Ahmad et al., 2016). It is well established that ZPR1 is severely downregulated in SMA (Ahmad et al., 2012; Doran et al., 2006; Genabai et al., 2015; Jiang et al., 2019; Kannan et al., 2020), but the biological significance of such down regulations is unclear. Like *SMN2*, ZPR1 is also a modifier protein (Ahmad et al., 2016). Overexpression of ZPR1 can lessen the severity of the SMA phenotype, which is already being explored for genetic therapy (Ahmad et al., 2016). Complete ZPR1 knockout in mice without SMN gene mutations results in axonal pathologies, like facial motor neuron degeneration, loss of motor neurons in the spinal cord, axon retraction, and disruption of microtubules, all contributing to neurodegeneration (Ahmad et al., 2012; Doran et al., 2006).

It is well known that high-fat diets can mediate cognitive decline (Pistell et al., 2010). But not all high-fat diet constituents behave the same; it is known that n-3 polyunsaturated fatty acids (PUFAs) benefit the brain by improving memory and mood and protecting against cognitive decline. In this article, a high-fat diet refers to large quantities of saturated fatty acids (SFA), trans fatty acids (TFA), and simple monosaccharides consumption over a long period (Cordain et al., 2005; Demigne et al., 2006). This type of diet contains little to no amount of PUFAs (Cordain et al., 2005; Demigne et al., 2006). Interestingly, Cifre et al. demonstrated ZPR1 downregulation and amyloid-beta precursor protein (APP) upregulation in the hippocampus (Cifre et al., 2018), as well as cognitive decline in rats fed with high-fat, isocaloric diets containing large amounts of saturated fats. These findings mentioned above, in combination with ZPR1's role in SMA pathology and its ability to participate in the basal transcription apparatus of the PPAR-γ gene, prompt us to review the role of ZPR1 protein in high-fat diet-mediated cognitive decline.

## 2 High-fat diet and cognitive decline

60–70% of all dementia comprises Alzheimer's disease (Barker et al., 2002). Several studies indicate that high-fat diets correlate with Alzheimer's disease. It is unknown how a high-fat diet causes dementia, but several hypotheses exist. Nägga et al. conducted a longitudinal cohort study beginning with cognitively healthy individuals. Twenty years later, those with high-lipid levels were independently associated with abnormal Aβ42 levels and Aβ42/p-tau ratio in cerebrospinal fluid, indicating Alzheimer's disease pathology (Nägga et al., 2018). In addition to a high-fat diet, metabolic syndrome, high plasma triglycerides, and T2DM also put individuals at a greater risk for developing Alzheimer's disease. Kim et al. conclude that patients with metabolic syndrome are 11 times more likely to develop Alzheimer's disease but not vascular dementia (Kim et al., 2021). However, other studies have linked vascular dementia to high plasma triglycerides (Raffaitin et al., 2009). One of the strongest genetic determinants of Alzheimer's disease is the allele Apolipoprotein E ε4 (APOEε4). Lui et al. hypothesize that lipidated ApoE binds to Aβ, enabling Aβ uptake through transmembrane receptors, like lipoprotein receptor-related protein 1 (LRP1), into glial cells (Liu et al., 2013). Isoform APOEε4 may affect this process by changing the amount of lipidation (Liu et al., 2013). APOE also interferes with the clearance of Aβ at the blood-brain barrier (Liu et al., 2013). Like APOE, ZPR1 also participates in lipid metabolism; it lies on chromosome band 11q23.3, and ZPR1 variant rs964184 has been consistently linked to high plasma triglyceride level, metabolic syndrome, T2DM, and NAFLD (Esteve-Luque et al., 2021; Fu et al., 2015; Gombojav et al., 2016; Guan et al., 2016; Mirhafez et al., 2016; Paquette et al., 2020). Several independent studies have repeatedly found the rs964184 variant among Latino, South and East Asian, and Caucasian populations (Esteve-Luque et al., 2021; Fu et al., 2015; Gombojav et al., 2016; Guan et al., 2016; Mirhafez et al., 2016; Paquette et al., 2020), reinforcing the notion that the effects of such polymorphisms may be universal.

## 3 ZPR1 in cognitive decline

### 3.1 ZPR1 and neuronal survival

In SMA, it was shown the downregulation of ZPR1 triggered the JNK signal cascade and activated Caspase 3 (Jiang et al., 2019), which causes the death of motor neurons; the authors did not mention death by ZPR1 downregulation in the hippocampus (Jiang et al., 2019). Cifre et al. showed upregulation of Caspase 3 and downregulation of ZPR1 caused by high-fat, isocaloric diet in 3-, 5-, and 6-month-old mice (Cifre et al., 2018). Wei et al. also demonstrated increased cleaved Caspase 3 in hippocampus tissues of aged mice on various diets (Wei et al., 2018). The level of cleaved caspase-3 was highest in high-fat sugar-fed mice, followed by high-fat diet mice, followed by ad libitum surgery diet mice, and the least amount of cleaved caspase-3 was detected in ad libitum control diet (Wei et al., 2018). These data indicate increased cleaved caspase-3 levels in high-fat diets in comparison to sugar and control groups.

ZPR1 binds to *SMN2* locus 5q13 to enhance transcription. *SMN2* gene is ubiquitously expressed and facilitates splicing in snRNP complexes, and it is definitively shown to be expressed in various parts of the hippocampus (Battaglia et al., 1997). Kannan et al. demonstrated that prolonged downregulation of ZPR1 causes less *SMN2* gene to be transcribed, producing less SMN protein, in turn downregulating the production of senataxin (SETX), a helicase, and DNA-activated protein kinase catalytic subunit (DNA-PKcs) (Kannan et al., 2018, Kannan et al., 2020). DNA-PKcs is a key component in non-homologous end joining (NHEJ) repair, which is an important repair mechanism for neurons (Kannan et al., 2018, Kannan et al., 2020). The lack of SETX promotes R-loops, and the lack of DNA-PCKcs reduces NHEJ repair (Kannan et al., 2018, Kannan et al., 2020).

R-loops are loops of partially coiled genetic material that contain two DNA strands and one RNA strand, indicating incomplete transcription (Kannan et al., 2018, Kannan et al., 2020). R-loops accumulate and are more likely to produce double-stranded breaks (DBS), creating genomic instability (Kannan et al., 2018, Kannan et al., 2020). These nuclear responses trigger cellular stress pathways, specifically the mixed-lineage protein kinase 3 (MLK3), mitogen-activated protein kinase 7 (MKK7), and c-Jun N-terminal kinase 3 (JNK3) (Jiang et al., 2019), (Kannan et al., 2018, Kannan et al., 2020). Lower concentrations of ZPR1 result in c-Jun phosphorylation, triggering cytochrome C release, and cleavage of caspase 3, resulting in neurodegeneration (Jiang et al., 2019, Kannan et al., 2018, Kannan et al., 2020). Downregulation of ZPR1 caused by high-fat diet can also follow this sequence of events leading to R-loop accumulation and reduction in NHEJ repair. Kannan et al. also show that the upregulation of ZPR1 in SMN mutated mice exhibited significantly higher levels of SETX, increased levels of DNA-PKcs, and a lower number of R-loops, thereby ameliorating the severity of the SMA phenotype (Kannan et al., 2018, Kannan et al., 2020).

**Fig. 1:**
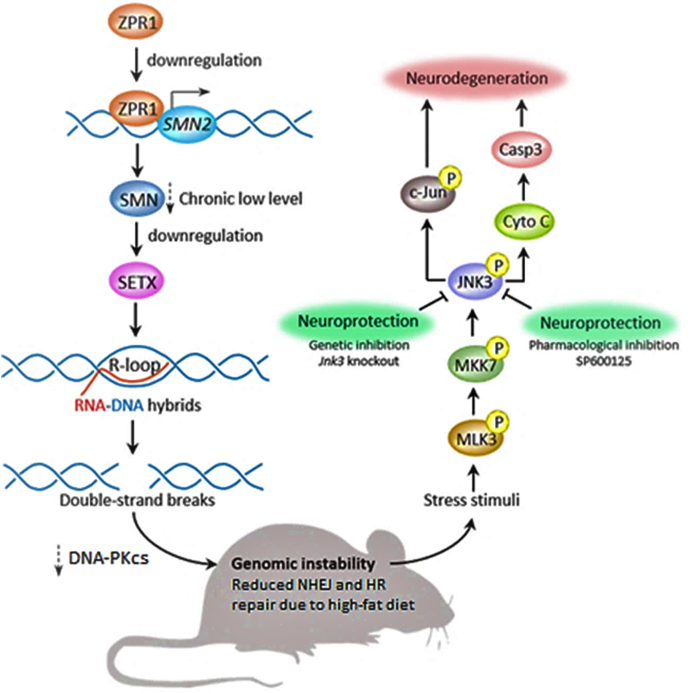
Schematic diagram summarizing ZPR1 mediated death of hippocampal neurons caused by high-fat diet. Image is adapted and modified from earlier publications (Jiang et al 2019; Kannan et al 2018; Kannan et al 2020).

Selective downregulation of ZPR1 can produce less SMN protein in the hippocampus but not in motor neurons, avoiding the SMA phenotype. The R-loops increase the chance of double-stranded breaks and cause genomic instability if the number of breaks outweighs homologous recombination (HR) and NHEJ repair. Yu et al. demonstrated a significant increase in double-stranded DNA breaks (DSBs) in 6 month-old mice, wild-type and APP/PSEN1 genotype mice, on a 60% lard diet and showed the HR repair process to be significantly diminished in hippocampal neurons based on the decreased number of RAD51-positive foci (Yu et al., 2018). RAD51-positive foci is a biomarker that is directly proportional to HR repair (Yu et al., 2018). NHEJ mediated repair was unchanged in wild-type mice on a high-fat diet but decreased in NHEJ repair in mice on a high-fat diet with APP/PSEN1 genotyped mice (Yu et al., 2018). These data demonstrate decreased DSB repair mechanisms in hippocampal neurons in mice on a high-fat diet, encouraging ZPR1 mediated neurodegeneration. Together, high-fat diet and ZPR1 downregulation drastically reduce NHEJ and HR repair, promoting genomic instability in the hippocampus. The reduction in HR repair caused by high-fat diet may cause the upregulation of Caspase 3, mediating the death of hippocampal neurons. Fig. 1 demonstrates the schematic overview of ZPR1-mediated degeneration of neurons caused by a high-fat diet.

### 3.2 ZPR1 in BDNF synthesis

BDNF and ZPR1 are indirectly related. ZPR1 unbinds from EGFR upon epidermal growth factor (EGF) binding. Then, ZPR1 relocates to the nucleus, binds to the promoter region of PPAR-γ 1/2, and partakes in the basal transcription apparatus for this gene (Corton et al., 2000; Kannan et al., 2020; Mangelsdorf et al., 1995). PPAR-γ is involved in hippocampal memory consolidation. Jahrling et al. showed that phosphorylated ERK2 (pERK2) forms a complex with PPAR-γ, CREB-binding protein (CBP), and mitogen-activated protein kinase (MEK) to transcribe cAMP response element-binding protein (CREB) (Jahrling et al., 2014). This complex binds to CRE and PPRE regions and is part of the basal transcription apparatus to transcribe CREB (Cifre et al., 2018). Of relevance, CREB is a transcription factor for the synthesis of BDNF. The quantity of generated PPAR-γ and pERK2 complexes influences the amount of CREB made and changes the hippocampal ratio of PPAR-γ and pERK2 (d’Angelo et al., 2019). Therefore, more ZPR1 could mean more BDNF by increasing PPAR-γ transcription, possibly preventing cognitive decline. Hence, ZPR1 and BDNF are related through PPAR-γ. In the tropomyosin receptor kinase B (Trk B) pathway, BDNF binds to Trk B due to its high affinity (Pradhan et al., 2019), activating the RAS/RAF/MEK/ERK2 pathway. ERK2, generated from Trk B cascade signaling, can participate in the PPAR-γ complex to regulate CREB transcription (Jacob et al., 2002). It is expected that concentrations of Trk B and BDNF are proportional, but Cifre et al. displayed no significant change in BDNF and CREB by isocaloric high-fat diets (Cifre et al., 2018). The investigators noted, “the expression of downstream effectors of BDNF action was reduced in the hippocampus of these animals, including Trk B, coding for BDNF receptor” (Cifre et al., 2018). Downregulation of ZPR1 directly influences the concentration of PPAR-γ and indirectly affects the ratio of pERK2 to PPAR-γ and regulation of CREB transcription. Decreased activation of the EGFR receptor has a similar effect to downregulation of ZPR1 because less ZPR1 translocates to the nucleus. Lim et al. showed that low plasma EGF levels correlated with cognitive decline in Alzheimer's disease and Parkinson's Disease patients, suggesting EGF as a biomarker for cognitive decline (Lim et al., 2016). The downregulation of ZPR1 due to a high-fat diet directly reduces the concentration of PPAR-γ, thereby resulting in less CREB and downstream less BDNF, resulting in less synaptic plasticity, which is needed for long term memory formation.

### 3.3 ZPR1 in PPAR-γ transcription

Experiments in mice supported the idea that PPAR-γ agonists improve hippocampal cognition (Denner et al 2012). Contrary, the PPAR-γ agonist did not improve cognition and memory function in human clinical trials with Alzheimer's disease patients (Becker and Greig 2013). Researchers hypothesize that PPAR-γ alone cannot ameliorate Alzheimer's disease memory loss (Jahrling et al 2014). But, when PPAR-γ agonist was administered to those with mild cognitive decline, hippocampal memory showed improved functionality (Risner et al 2006; Sato et al 2011; Stockhorst et al 2004; Watson et al 2005). Pharmacological intervention with retinoid X receptor (RXR) activation and PPAR-γ agonist together improved cognition in Alzheimer's disease patients (Yamanaka et al 2012). Jahrling et al. demonstrated PPAR-γ forming a complex with pERK2, MEK, and CBP to transcribe CREB protein, a protein necessary for neuroplasticity and long-term memory formation (Jahrling et al 2014). The investigators only observed the formation of PPAR-γ/pERK2 complex in human hippocampi with Alzheimer's disease and mice hippocampi with Alzheimer's disease, not in the control hippocampi of humans and mice (Jahrling et al 2014). The PPAR-γ/pERK2 ratio correlated positively with cognitive performance, quantified by Mini-Mental State Examination (MMSE) score, only in Alzheimer's disease affected hippocampi (Jahrling et al 2014). Jahrling et al. suggest the PPAR-γ/pERK2 ratio to be a compensation mechanism to restore disrupted ERK signaling in Alzheimer's pathogenesis (Jahrling et al 2014). Of importance, ZPR1 binds to the promoter of the PPAR-γ 1/2 gene (Corton et al., 2000, Kannan et al., 2020, Mangelsdorf et al., 1995), thereby influencing the concentration of PPAR-γ available to partake in such complexes.

The effect of ZPR1 binding to the PPAR-γ 1/2 gene has several repercussions, depending on cell type. The general structure of the PPAR gene contains the following functional domains: A/B, C, D, and E/F. Of interest, the C domain, also called the DNA-binding domain (DBD), codes for a highly conserved region of DNA that binds to two zinc finger proteins (Corton et al 2000; Mangelsdorf et al 1995). For PPAR-γ 1/2, this C domain binds to ZPR1 (Corton et al 2000; Mangelsdorf et al 1995). The effects of ZPR1 binding to the PPAR-γ 1/2 gene include, but are not limited to, promoting insulin sensitivity and anti-inflammatory activation. PPAR-γ 1/2 plays a key role in insulin sensitivity, fat distribution, adipogenesis, and obesity (Tsai and Maeda 2005). Previous studies have linked ZPR1 polymorphism rs964184 to onset of and complications from T2DM (Esteve-Luque et al 2021; Fu et al 2015; Gombojav et al 2016; Guan et al 2016; Mirhafez et al 2016; Paquette et al 2020). This polymorphism may have altered the primary structure of ZPR1, affecting its ability to bind to PPAR-γ gene. If less ZPR1 binds to the promotor of PPAR-γ, then a reduced amount of PPAR-γ protein is produced in adipocytes within the subcutaneous tissue; such dysregulation discourages adipogenesis, resulting in higher levels of fatty acid circulation in the blood, encouraging visceral lipid accumulation over peripheral lipid accumulation (Tsai and Maeda 2005). On a smaller level, the effect of ZPR1 binding to the PAPR-γ gene can be observed in diabetic ulcers. Takematsu et al. studied gene expression changes in skin samples taken from patients with and without T2DM (Takematsu et al 2020). They demonstrated significant downregulation of ZPR1 mRNA in skin samples from T2DM patients (Takematsu et al 2020). Mirza et al. demonstrated that inflammatory cytokine IL-β1 suppressed PPAR-γ activity in mouse and human macrophages, prolonging wound healing (Chen et al 2015; Mirza et al 2015). Likewise, macrophage PPAR-γ knockout mice had extended inflammation, delayed wound closure, and decreased growth factor activity (Chen et al 2015; Mirza et al 2015). Topical PPAR-γ agonists promoted the healing of ulcers in mice (Chen et al 2015; Mirza et al 2015). These findings are expected since many researchers have shown PPAR-γ could promote anti-inflammatory reactions in macrophages. The formation of diabetic skin ulcers relies on the reduction of PPAR-γ and ZPR1. The increase in ZPR1 may lead to an increase in PPAR-γ, accelerating wound healing. This example is important because it illustrates the most direct and clear effect of ZPR1 binding to the PPAR-γ gene.

### 3.4 ZPR1 and high-fat diet relationship

**Fig. 2:**
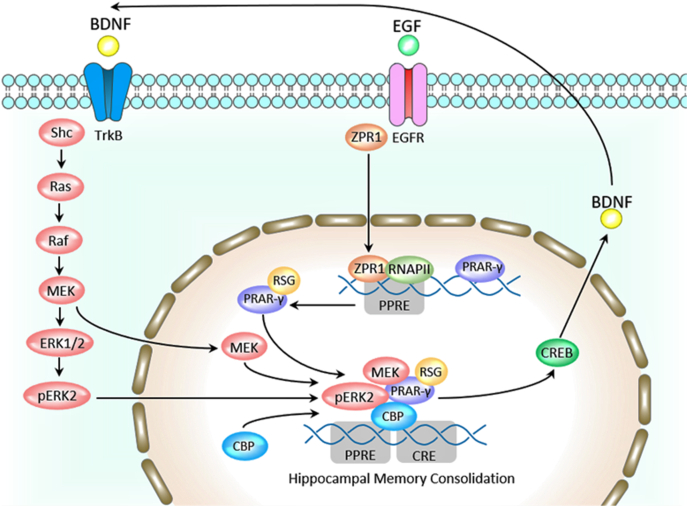
Schematic diagram showing the cyclical relationship between ZPR1, Trk B, CREB, and BDNF. Image is modified from earlier publications (Pradhan et al 2019; Jahrling et al 2014; Andero et al 2014).

Downregulation of ZPR1 indirectly affects brain-derived neurotrophic factor (BDNF) and directly affects the ratio of PPAR-γ/pERK2 (Fig. 2). BDNF supports neuron health, differentiation, growth, and synapse formation (Cunha et al 2010). Upon binding to its receptor, BDNF elicits downstream effects such as releasing Ca2+ from intracellular stores, transcription of CREB, activation of NF-κB, and inducing the release of glutamate and gamma-aminobutyric acid (GABA) into synapses (Cunha et al 2010). Cifre et al. showed the downregulation of ZPR1 and Trk B mRNA expression in rat hippocampal cells; the level of ZPR1 mRNA was significantly reduced in the hippocampus of 6-month-old rats on a 45% high-fat, isocaloric diet when compared to the control group (Cifre et al 2018). ZPR1 mRNA expression was also significantly reduced in 5 months and 3-months-old rats on a 60% high-fat, isocaloric diet compared to the control group (Cifre et al 2018). ZPR1 mRNA negatively correlated with the HOMA-IR index. Likewise, Trk B and BDNF levels were significantly reduced in the hippocampus of psychosocially stressed, obese mice (Agrimi et al 2019). Trk B correlated negatively with the HOMA-IR index in the hippocampus of the 60% high-fat group (Cifre et al 2018). The converse is also true: cholesterol loss in aged mice significantly correlated with increased Trk B activity, measured by levels of phosphorylation in hippocampal neurons (Martin et al 2008). Cholesterol increases in the plasma membranes of aged hippocampal neurons significantly reduced Trk B activity, illustrating that excess amounts of lipids suppress Trk B phosphorylation (Martin et al 2008). Additionally, Kalivarathan et al. demonstrated downregulation of BDNF expression in the hippocampus of rats on high-fat, high-fructose diets (Kalivarathan et al 2017). In fact, numerous investigators have shown downregulation of hippocampal BDNF in high-fat diet rodent models. Finally, Huang et al. used q-PCR to demonstrate, “the down-regulation of hippocampal AMP-activated protein kinase (AMPK) and CREB expression levels in the high-fat diet-fed mice” (Huang et al 2021). Together, these data support the downregulation of ZPR1, Trk B, BDNF, and CREB due to a high-fat diet in adult murine models. The downregulation of ZPR1 due to high-fat diet may cause a decrease in BDNF, resulting in dysfunctional learning and memory formation. Previously, Nogusa et al. demonstrated the upregulation of ZPR1 in mice hippocampus due to high-fat diet in 11-week-old mice (Nogusa et al 2006). Cifre et al. acknowledge this increase in ZPR1 and explained that the increase could be due to the younger age of the mice (Cifre et al 2018). Since ZPR1 is sent to the nucleus upon mitogen activation, growth upregulates the concentration of ZPR1 to promote cellular proliferation.

## 4 Conclusion

Long-term molecular changes in ZPR1 can lead to neurodegenerative disease based on the above-mentioned sequence of events in the hippocampus. This is a consequence of ZPR1 downregulation, occurring in later life, contributing to various forms of dementia triggered by a high-fat diet. Direct evidence of manipulation of ZPR1 on cognitive function outside of SMA can be seen in a patient with ZPR1 syndrome. The investigators observed a novel, recessive, missense ZPR1 mutation in the hydrophobic core of the protein (Ito et al 2018). Proteosomes degraded the misfolded ZPR1 protein, therefore there was little to no concentration of ZPR1 in fibroblasts from the patient; this phenomenon could apply to other cells (Ito et al 2018). The patient exhibited “moderate intellectual disability,” as well as optic nerve atrophy, sensorineural hearing loss, microcephaly, and many other multisystemic signs and symptoms (Ito et al 2018). These human data show that ZPR1 is needed for CNS development and cognitive function, independent of SMA.

This brief article focuses on how the downregulation of ZPR1 by a high-fat diet can contribute to cognitive decline by directly influencing the concentration of PPAR-γ and indirectly reducing the concentration of BDNF to decrease neural plasticity. Downregulation of ZPR1 in the hippocampus, and not in motor neurons, can directly compromise hippocampal function. Low levels of SMN2 transcription over a long period led to a reduction of SETX and DNA-PKcs, which adversely affects NHEJ repair. In combination with the further reduction of HR and NHEJ repair mechanisms due to high-fat diet, this encourages the formation of R-loops and DSBs, resulting in genomic instability, activating the JNK signal cascade. Ultimately, the JNK signal cascade produces an increased concentration of cleaved Caspase 3. Given that the modality of these events is limited to hippocampal neurons, the death of motor neurons and multisystemic splicing defects are not present. It is no surprise that ZPR1 levels are affected due to a high-fat diet since human studies with ZPR1 variant rs964184 show it to be involved with plasma triglycerides, metabolic syndrome, and T2DM, reinforcing the idea that this protein is involved in lipid metabolism. More experimental studies are needed to further understand how ZPR1 downregulation, a key molecular event, connects a high-fat diet to the eventual cognitive decline.

Two important gaps in this topic are the lack of research in humans and the absence of intervention studies. Researchers can look at brain tissues from patients diagnosed with Alzheimer's disease, insulin resistance, and obesity and measure the expression levels of ZPR1. Once the proposed idea is validated in humans, further intervention studies can be designed with the aim to increase levels of ZPR1 in the hippocampus during the early stages of diseases to determine the possible therapeutic benefits.

## Author contributions

**Mythri Chittilla**: collected information and drafted the manuscript. **Nuraly S. Akimbekov**: edited the manuscript and contributed to the artworks. **Mohammed S. Razzaque**: conceptualized and reviewed the manuscript.

## Declaration of competing interest

The authors declare that they have no known competing financial interests or personal relationships that could have appeared to influence the work reported in this paper.
